# Timely Intervention in Light Chain Cardiac Amyloidosis

**DOI:** 10.1016/j.jaccas.2025.104809

**Published:** 2025-08-13

**Authors:** Annas Rahman, Firas Anaya, Bala Pushparaji, Phoo Pwint Nandar

**Affiliations:** Division of Cardiovascular Medicine, Heart and Vascular Institute, MetroHealth Medical Center/Case Western Reserve University, Cleveland, Ohio, USA

**Keywords:** cardiomyopathy, diastolic heart failure, echocardiography, imaging, restrictive, right-sided catheterization, strain rate imaging

## Abstract

**Background:**

Light-chain cardiac amyloidosis (AL-CA) is often overlooked and misdiagnosed as simple diastolic dysfunction, which can lead to worse outcomes if an accurate diagnosis is delayed.

**Case Summary:**

This case highlights the importance of utilizing a multidisciplinary team approach and multimodality imaging, such as cardiac magnetic resonance and endomyocardial biopsy, in the prompt diagnosis and treatment of AL-CA with a tailored hematologic treatment regimen in a 70-year-old male. Patient achieved complete remission with Dara-CyBorD (daratumumab, cyclophosphamide, bortezomib, and dexamethasone) therapy in 5 months, demonstrating stabilization and potential reversal of myocardial dysfunction.

**Discussion:**

Of the many causes of heart failure with preserved ejection fraction, AL-CA can have an especially poor prognosis if diagnosis is delayed. It is crucial to be aware of the clinical presentation of AL-CA, the wide variety of diagnostic tools, multidisciplinary management, and intensive treatment options available, and importance of regular follow-up.

**Take-Home Messages:**

Recognizing the hallmark diagnostic features of AL-CA is crucial for differentiating it from other causes of diastolic dysfunction, particularly in the early work-up period. Timely diagnosis of AL-CA and assessment of the infiltrative burden are essential for selecting an individualized hematologic treatment course.

## Graphical Abstract

**Figure:**
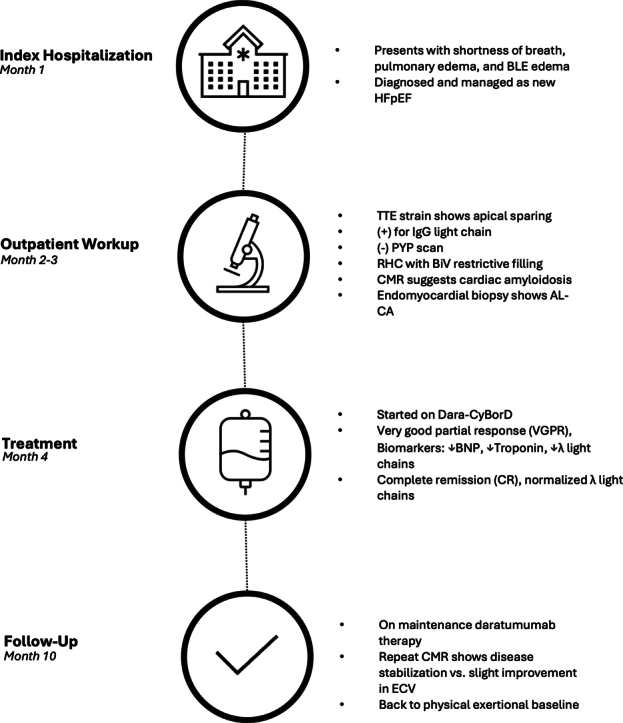

## History of Presentation

A 70-year-old man presented with 1 week of breathlessness, productive cough, lethargy, and edema of the abdomen and bilateral lower extremities. On presentation, he was saturating 87% on room air, B-type natriuretic peptide (BNP) was 732 pg/mL, and high-sensitivity troponin I (HST) was elevated to 124 ng/L. Chest x-ray film revealed patchy alveolar opacities in the left lower lobe, an enlarged cardiac silhouette compared to previous imaging, and new small bilateral pleural effusions.

## Past Medical History

The patient has a past medical history of dyslipidemia, coronary artery disease requiring a drug-eluting stent to his mid left anterior descending artery in 2014 for a 90% stenotic lesion; obstructive sleep apnea for which he uses continuous positive airway pressure; hyperparathyroidism, which required a partial parathyroidectomy in 2016; and erectile dysfunction. His history is also notable for a 25-pack-year smoking history but quit in 1993. On family history, he reported multiple first- and second-degree relatives who required coronary revascularization in their 50s.

## Differential Diagnosis

A broad consideration for the causes of dyspnea and generalized edema on physical examination can include pneumonia, chronic pulmonary disease (eg, chronic obstructive pulmonary disease, interstitial lung disease), pulmonary embolism, nephrotic syndrome, and cirrhosis. Focused cardiac etiologies include an acute coronary syndrome, left- or right-sided heart failure, active pericardial process (eg, pericarditis, tamponade), valvular dysfunction, or pulmonary hypertension.

## Investigations

**Figure 1:**
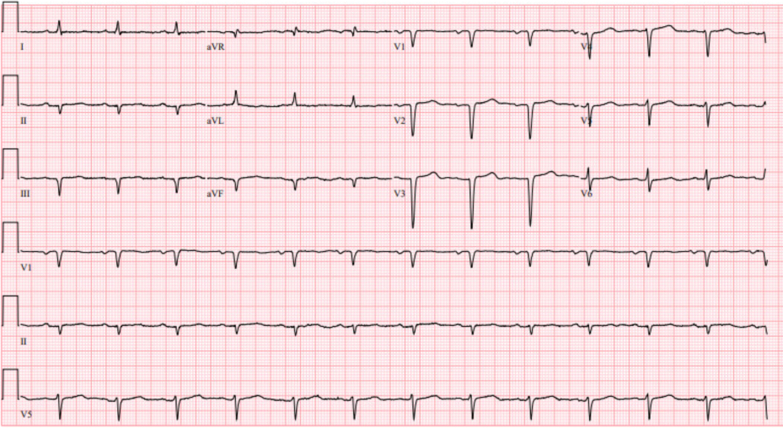
Electrocardiography at Index Hospitalization Electrocardiogram reveals a low-voltage pattern.

An electrocardiogram showed normal sinus rhythm, low voltage across limb and precordial leads, and Q waves in the inferior leads (Figure 1). Transthoracic echocardiography (TTE) revealed a left ventricular ejection fraction (LVEF) of 50% and a small pericardial effusion. The patient was determined to have a troponin elevation from a type 2 myocardial infarction related to a volume overloaded state and new heart failure with preserved ejection fraction. The patient underwent intravenous diuresis with improvement to euvolemia and was discharged with oral metoprolol, lisinopril, spironolactone, and empagliflozin.

**Figure 2:**
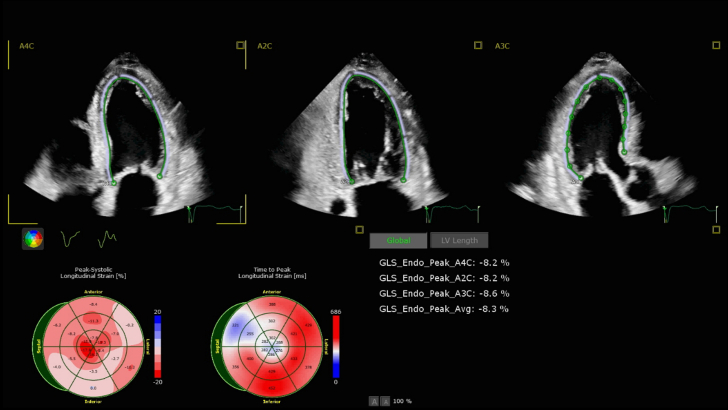
Transthoracic Echocardiography Strain From First Hospitalization Global longitudinal strain is reduced.

At his outpatient cardiology follow-up, a repeat assessment of the inpatient TTE showed global longitudinal strain (GLS) of −8.1%, a relative regional strain ratio of 1.21, and an LVEF-to-strain ratio >4, all highly suspicious for cardiac amyloidosis (Figure 2). Amyloid labs including kappa and immunofixation electrophoresis were positive for IgG kappa light-chain monoclonal gammopathy of undetermined significance. Further imaging with a pyrophosphate scan was negative for uptake, and thus for transthyretin amyloidosis. Later, a bone marrow biopsy with hematology-oncology revealed 9% monoclonal plasma cells, at least compatible with monoclonal gammopathy of undetermined significance.

The patient then underwent right heart catheterization and endomyocardial biopsy. The right heart catheterization showed an increased biventricular filling pattern and a low cardiac index (Supplemental Figure 1), right ventricular endomyocardial biopsy confirmed amyloid deposition with tissue typing, and mass spectrometry showed light-chain amyloidosis (AL-CA). Subsequent prechemotherapy cardiac magnetic resonance (CMR) showed an LVEF of 49% and late gadolinium uptake in the basal and mid endocardial distributions strongly suggestive of cardiac amyloidosis (CA), as well as an extracellular volume (ECV) of 53%.

## Management (Medical/Interventions)

After consultation with hematology-oncology, the patient was started on combination therapy with Dara-CyBorD (daratumumab, cyclophosphamide, bortezomib, and dexamethasone). After 2.5 months, laboratory results indicated hematologic very good partial response (VGPR) (Table 1), and by 5 months he achieved complete remission (CR). He was started on maintenance daratumumab therapy for 18 cycles and remained in CR 3 months later.

**Table 1:** Laboratory Markers Over Time in Management of AL-CA

	BNP (pg/mL)	HST (ng/L)	IgG (mg/dL)	Lambda FLC (mg/L)
Pretreatment	732	124	756	191
Month 1	738	152	550	76
Month 2	523	81	442	62
Month 8	513	34	359	12

AL-CA = light-chain cardiac amyloidosis; BNP = B-type natriuretic peptide; FLC = free light chain; HST = high-sensitivity troponin I.

## Outcome and Follow-Up

After 1 month of treatment, the patient showed biomarker response with decrease in BNP, HST, and kappa light chain. By 3 months, he had VGPR, and by month 5 his kappa levels normalized and he had CR.

He was started on maintenance daratumumab therapy at month 6 and at the time of writing this paper had been on it for 3 months. Recent follow-up CMR shows stable LVEF, and stability vs slight improvement in his ECV compared with prior. He returned to his exertional baseline compared with 1 year before diagnosis.

## Discussion

CA remains frequently overlooked due to its similar clinical features to other cardiomyopathies, particularly heart failure with preserved ejection fraction and hypertrophic cardiomyopathy.^1^ The median time from symptom onset to diagnosing CA is nearly 2 years, a consequential delay especially for patients with AL-CA in whom survival after diagnosis can be as short as 4 months.^2^ The patient in our case was diagnosed within 2 months of symptom onset, a diagnostic success that enables earlier treatment and improved outcomes.

Over the last decade, significant advancements in multimodal imaging, biomarker-based risk stratification, and new therapeutic agents have improved early recognition and disease staging, which is crucial for initiating timely management. The American College of Cardiology Expert Consensus on Cardiac Amyloidosis stresses the importance of a multidisciplinary approach to CA. The American College of Cardiology also recommends broader screening in patients with unexplained heart failure, incorporating serum-free light-chain assays, immunofixation electrophoresis, and N-terminal pro-BNP testing.^3^

Diagnostic modalities include echocardiography findings, such as increased left ventricular wall thickness and diastolic dysfunction, should be alarming for CA. In particular, GLS with apical sparing is a hallmark feature that differentiates CA from hypertrophic cardiomyopathy. In a case-control study done at Cleveland Clinic, Phelan et al^4^ concluded that a relative apical longitudinal strain of 1.0, defined using the equation [average apical LS/(average basal LS + mid-LS]), was sensitive (93%) and specific (82%) in differentiating CA from control subjects. The logistic regression multivariate analysis showed that the relative apical LS was the only parameter predictive of CA (*P* = 0.004). However, CMR has emerged as the gold standard for noninvasive diagnosis, particularly through late gadolinium enhancement and ECV quantification >40% is strongly indicative of cardiac amyloid infiltration.^2^ In our case, the TTE showed GLS of −8.1% and left ventricular strain ratio of 1.21, both highly suspicious for CA. CMR showed an LVEF of 49% and late gadolinium update in the basal and mid endocardial distributions, which are strongly suggestive of CA, as well as an ECV of 53%.

The Mayo Clinic staging system, which incorporates N-terminal pro-BNP and cardiac troponins, remains a cornerstone of risk stratification in AL-CA.^5^ However, Fontana et al^2^ discussed how incorporating imaging-based metrics, such as positron emission tomography–based quantification of amyloid burden, provides superior prognostic accuracy. The American College of Cardiology consensus supports serial biomarker monitoring in conjunction with imaging modalities for disease staging and to assess treatment response.^3^ As recent literature suggests, reductions in light chain burden can improve myocardial function before amyloid regression occurs.^6^ This highlights the value of serial biomarker monitoring.

Despite advancements in noninvasive diagnostics, histologic confirmation remains necessary in cases of diagnostic uncertainty. Congo red staining, immunohistochemistry, and laser capture mass spectrometry remain the gold standard for differentiating AL-CA from transthyretin amyloidosis.^7^

Historically, autologous stem cell transplantation (ASCT) was considered the mainstay of treatment, but high transplant-related mortality (10%-20%) has limited its use.^7^ On the other hand, daratumumab, a CD38 monoclonal antibody, has since emerged as the first-line therapy, demonstrating superior hematologic response rates. Chung et al^8^ showed that nearly 80% of patients achieved VGPR or CR with daratumumab-based therapy. American College of Cardiology guidelines support daratumumab as the preferred induction therapy, particularly for patients with advanced cardiac involvement who are ineligible for ASCT that is significant cardiac involvement and poor functional status.^3^ In our case, given the mortality risk and patient's cardiac involvement, the decision was to forgo ASCT. The patient was initiated on Dara-CyBorD, achieving VGPR within 2 months and CR by 5 months, with normalized kappa light chains. Maintenance therapy with daratumumab was continued, with plans for 18 cycles. The treatment course was complicated by fatigue but otherwise was well tolerated, with significant improvement in cardiac biomarkers (BNP trended down from 732 to 523 pg/mL) and imaging findings. Repeat CMR showed stable to slightly improved ECV, though LVEF decreased slightly, reflecting the progressive nature of CA despite hematologic remission.

Emerging therapy for AL-CA includes venetoclax, a BCL-2 inhibitor, has demonstrated a good hematologic responses in patients with t(11;14) translocations.^9^ Additionally, novel antifibril antibodies such as CAEL-101 and birtamimab are under investigation for their potential to clear amyloid fibrils.^2^

The importance of multispecialty follow-up is highlighted in cases such as this. hematology-oncology oversaw the immuno- and chemotherapeutic regimen. At the same time, the patient maintained close cardiology follow-up for issues such as diuretic titration and arrhythmia monitoring, for which he was started on a very low beta-blocker dose after cardiac monitoring showed nonsustained ventricular tachycardia.

Finally, we should be mindful that heart failure management in AL-CA requires a tailored approach distinct from conventional heart failure with preserved ejection fraction treatments. Afterload-reducing agents part of GDMT, such as angiotensin-converting enzyme inhibitors and angiotensin receptor blockers, are often poorly tolerated in CA patients due to orthostasis from dysautonomia. Additionally, patients with restrictive cardiomyopathies rely on heart rate to maintain their stroke volumes, making them less tolerant to high-dose beta-blockers, which blunt heart rate response during exertion. In all, these unique features of CA patients necessitate alternative treatment approaches such as using diuretics and sodium-glucose cotransporter-2 inhibitors, which have demonstrated benefits in improving cardiac function without exacerbating hypotension.^3^

## Conclusions

CA, once thought to be a rare disease, now benefits from increased awareness among clinicians and cardiologists. This case highlights the importance of early diagnosis and intervention using a multidisciplinary approach involving cardiology, hematology, and advanced imaging in managing light-chain amyloidosis. The patient's response to Dara-CyBorD therapy highlights the potential for stabilization and even possible reversal of myocardial dysfunction when treatment is initiated promptly. Continued follow-up will be essential to assess long-term outcomes and the potential role of emerging therapies, such as venetoclax,^3^ in this high-risk population.

## Funding Support and Author Disclosures

The authors have reported that they have no relationships relevant to the contents of this paper to disclose.

**Take-Home Messages**

• Recognizing the hallmark diagnostic features of AL-CA is crucial for differentiating it from other causes of diastolic dysfunction, particularly in the early work-up period.
• Timely diagnosis of AL-CA and assessment of the infiltrative burden are essential for selecting an individualized hematologic treatment course.

**Visual Summary:**
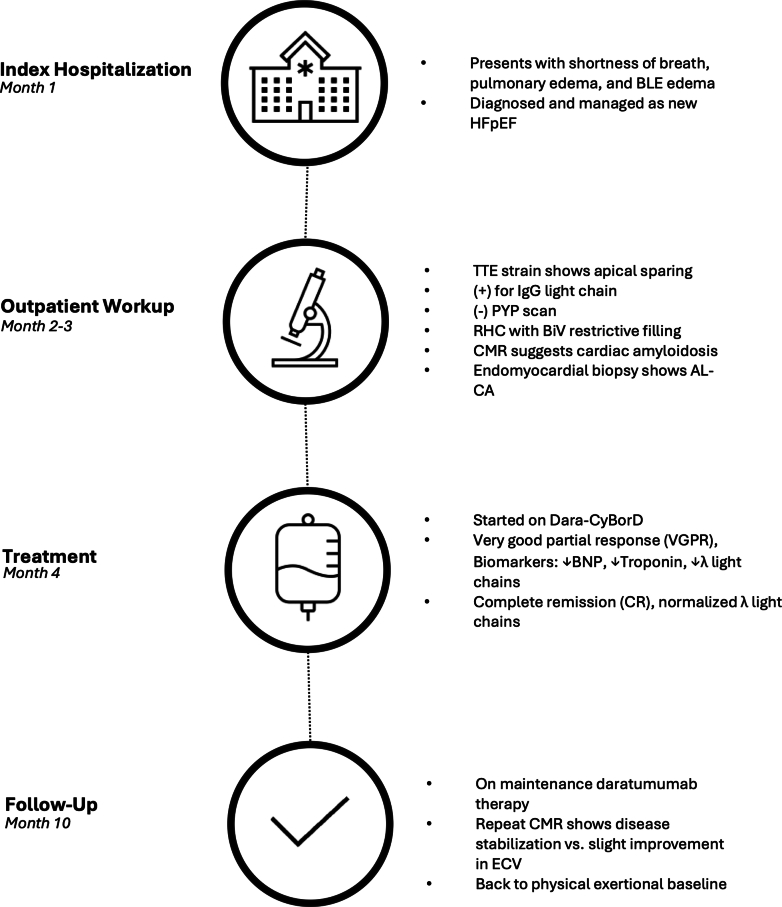
Patient Clinical Course
