# Supplementary figures and images for: Timely Intervention in Light Chain Cardiac Amyloidosis
Source: JACC Case Rep. 2025 Aug 13;30(23):104809. doi: 10.1016/j.jaccas.2025.104809 (PMC12462088; doi:10.1016/j.jaccas.2025.104809)

*Supplemental Figure 1. RIGHT-HEART CATHETERIZATION TRACINGS*

*
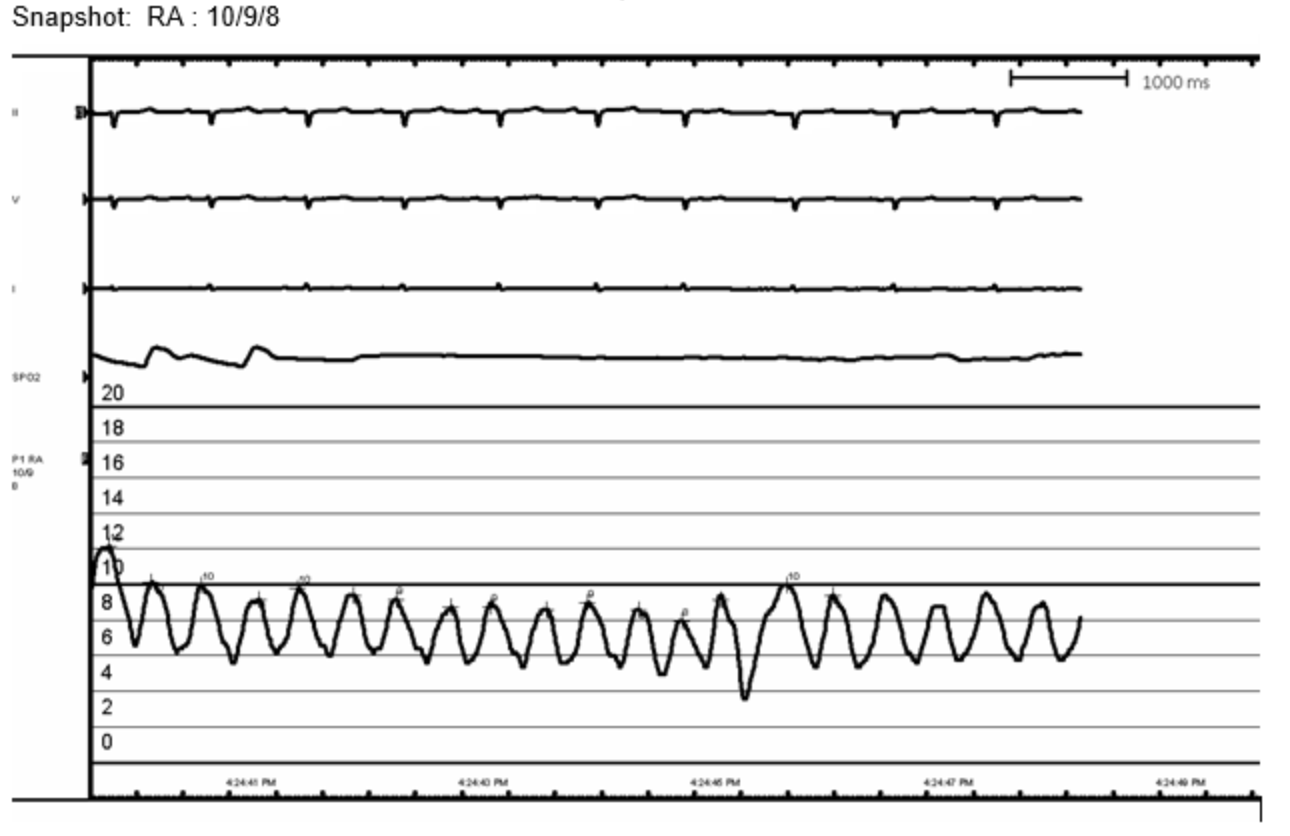

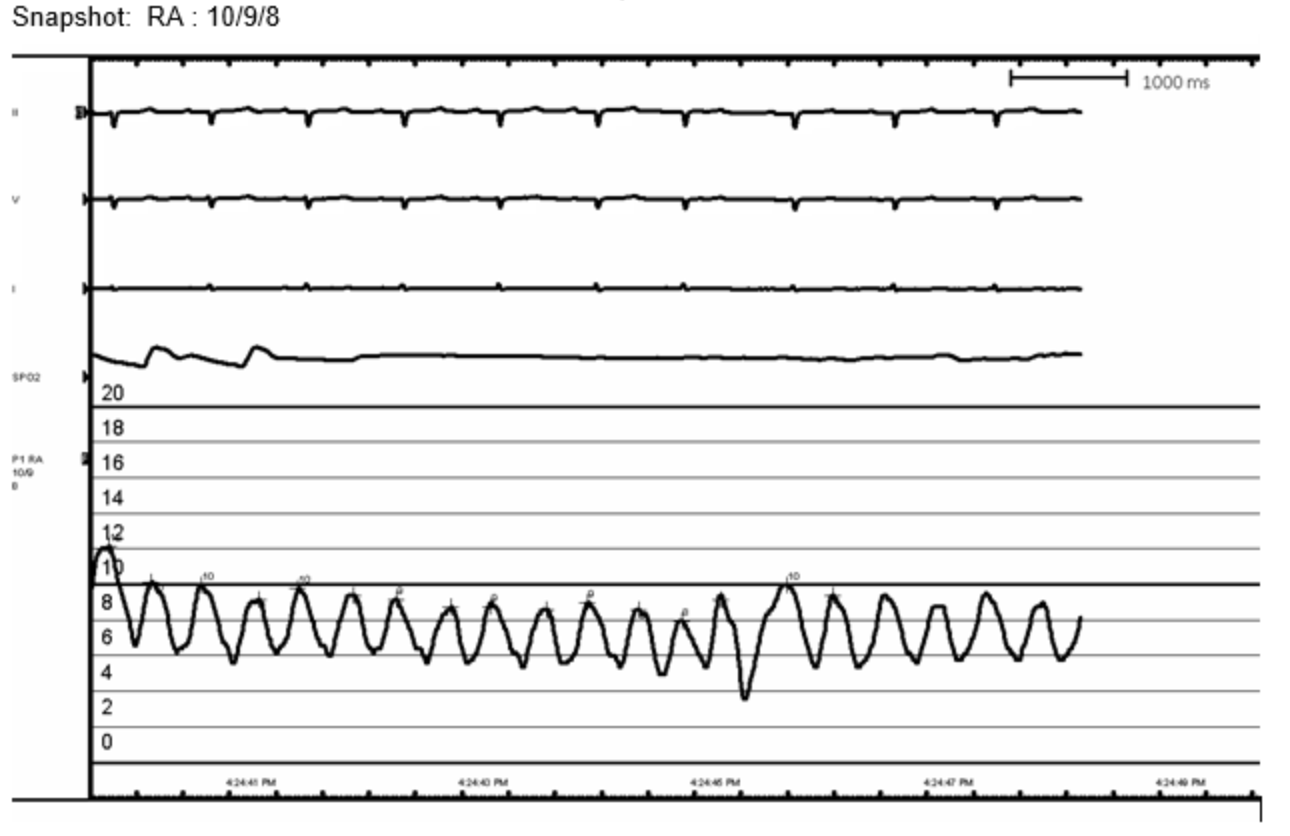
*

RA

RA

**
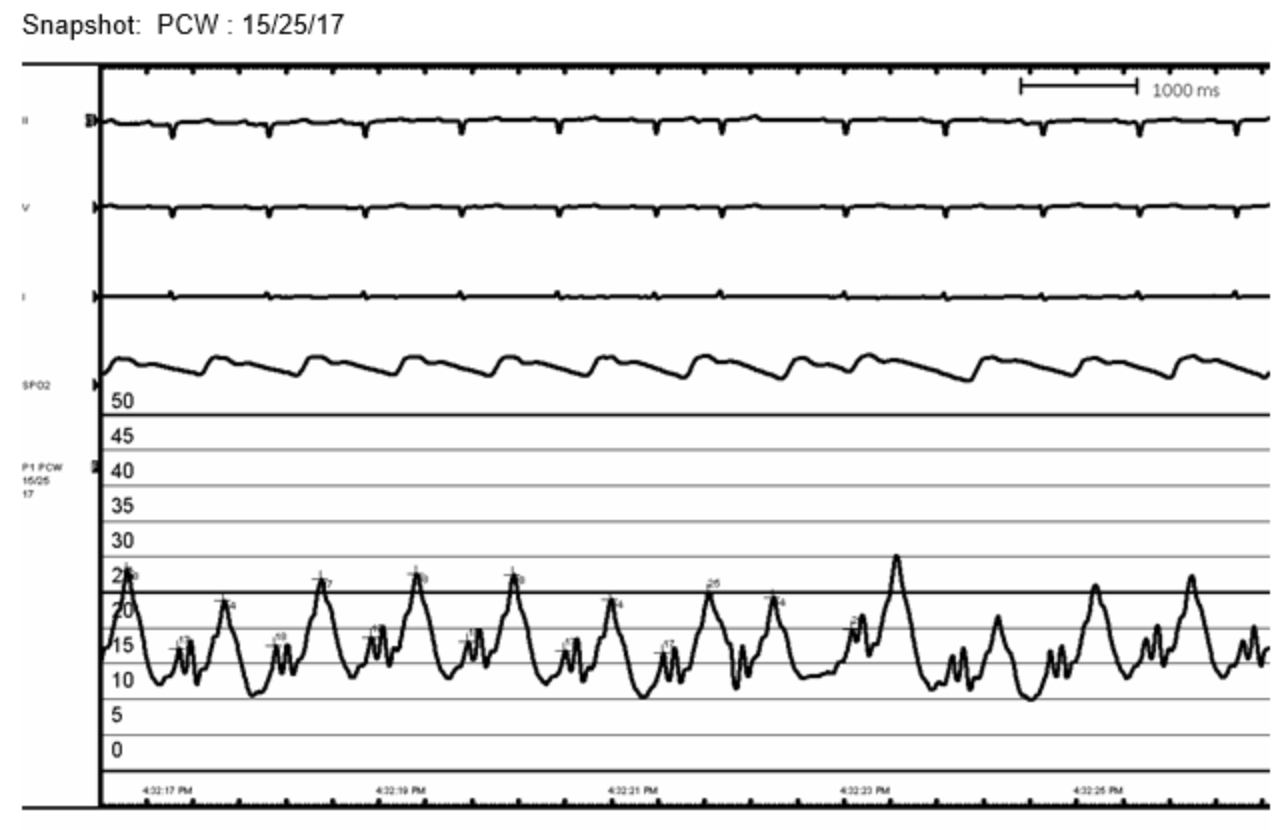

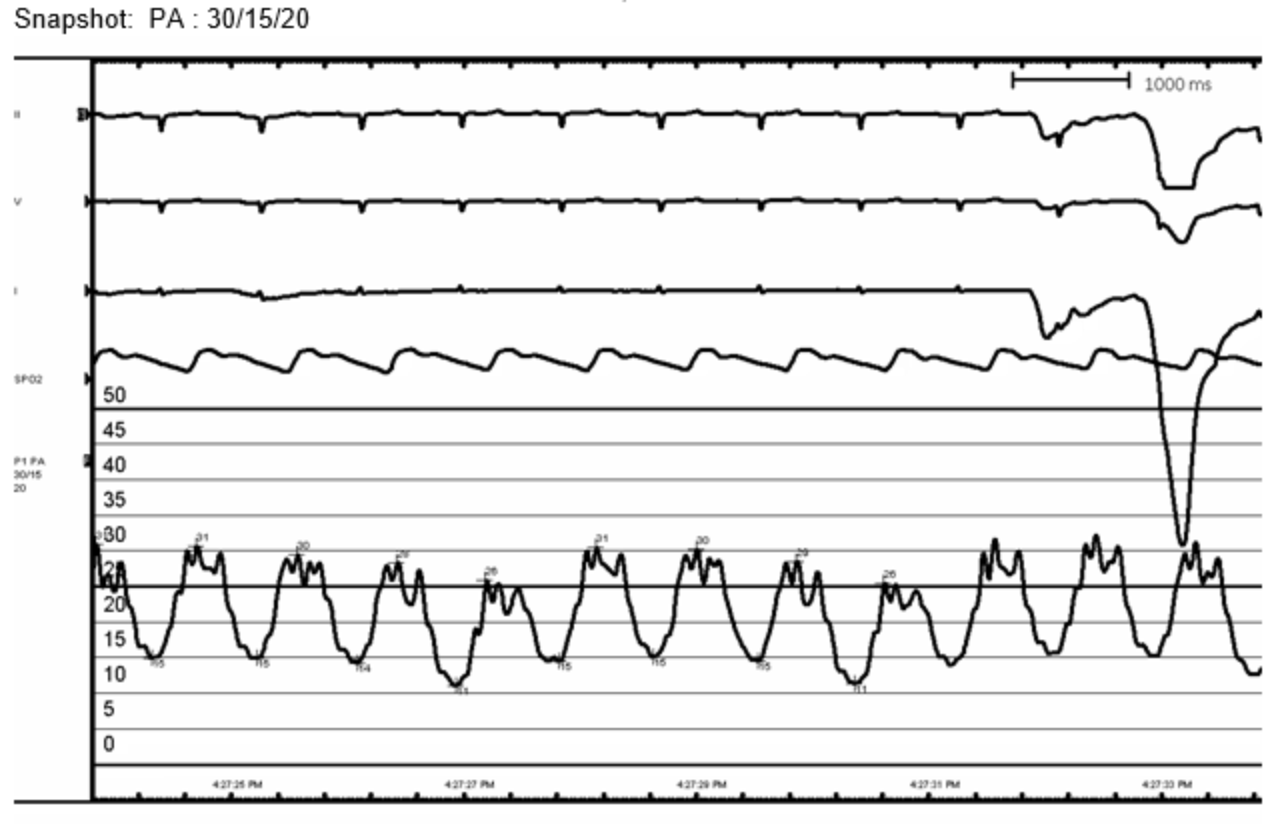
**

PCW

PA

Supplement: Supplemental Figure 1 [file mmc1.docx]
